# Simulation on the internal structure of three-dimensional proximal tibia under different mechanical environments

**DOI:** 10.1186/1475-925X-12-130

**Published:** 2013-12-20

**Authors:** Juan Fang, He Gong, Lingyan Kong, Dong Zhu

**Affiliations:** 1 Department of Engineering Mechanics, Nanling Campus, Jilin University, No. 5988 Renmin Street, Changchun 130025, People’s Republic of China; 2 Department of Orthopedic Surgery, No. 1 Hospital of Jilin University, Changchun 130021, People’s Republic of China

**Keywords:** Bone remodeling, Three-dimensional, Proximal tibia, Finite element, Valgus

## Abstract

**Background:**

Bone can adjust its morphological structure to adapt to the changes of mechanical environment, i.e. the bone structure change is related to mechanical loading. This implies that osteoarthritis may be closely associated with knee joint deformity. The purposes of this paper were to simulate the internal bone mineral density (BMD) change in three-dimensional (3D) proximal tibia under different mechanical environments, as well as to explore the relationship between mechanical environment and bone morphological abnormity.

**Methods:**

The right proximal tibia was scanned with CT to reconstruct a 3D proximal tibia model in MIMICS, then it was imported to finite element software ANSYS to establish 3D finite element model. The internal structure of 3D proximal tibia of young normal people was simulated using quantitative bone remodeling theory in combination with finite element method, then based on the changing pattern of joint contact force on the tibial plateau in valgus knees, the mechanical loading was changed, and the simulated normal tibia structure was used as initial structure to simulate the internal structure of 3D proximal tibia for old people with 6° valgus deformity. Four regions of interest (ROIs) were selected in the proximal tibia to quantitatively analyze BMD and compare with the clinical measurements.

**Results:**

The simulation results showed that the BMD distribution in 3D proximal tibia was consistent with clinical measurements in normal knees and that in valgus knees was consistent with the measurement of patients with osteoarthritis in clinics.

**Conclusions:**

It is shown that the change of mechanical environment is the main cause for the change of subchondral bone structure, and being under abnormal mechanical environment for a long time may lead to osteoarthritis. Besides, the simulation method adopted in this paper can more accurately simulate the internal structure of 3D proximal tibia under different mechanical environments. It helps to better understand the mechanism of osteoarthritis and provides theoretical basis and computational method for the prevention and treatment of osteoarthritis. It can also serve as basis for further study on periprosthetic BMD changes after total knee arthroplasty, and provide a theoretical basis for optimization design of prosthesis.

## Background

Osteoarthritis is a chronic joint disease, and severe osteoarthritis can cause knee-joint pain or malfunction. Total knee arthroplasty is needed to restore its function in serious cases. Its incidence and prevalence are rising with aging. It has serious influence on the life quality of the elder [1]. The typical radiographic features of knee osteoarthritis include degeneration of cartilage, subchondral bone sclerosis and osteophyte formation. It is thought that subchondral bone changes after cartilage degenerates, so much attention is paid to the change of cartilage and the treatment of osteoarthritis. However, recent studies showed that subchondral bone plays an important role in the development of osteoarthritis. The subchondral bone changes in the early stage of osteoarthritis and leads to the degeneration of articular cartilage, which may be the initial cause of osteoarthritis [2,3]. So it is important to investigate the roles of subchondral bone for the prevention and treatment of osteoarthritis.

In many cases, mechanical loading on subchondral bone appears to play a crucial role in the changes of BMD distribution of subchondral bone since bone tissue is living material that has the functionality to adapt to mechanical loading in terms of its mass and architecture. This attribute is known as functional adaptation, and it includes both modeling and remodeling processes [4]. Bone modeling happens simultaneously with bone growth, and its primary role is to control the structure and shape of bone during the growing period. Bone remodeling always exists in the whole life and its primary role is to renew and adjust the bone structure to adapt to the mechanical loading change [5]. The numerical simulation of bone functional adaptation behavior showed that the adaptation behaviors of bone tissue at different mechanical levels were different and mechanical stimulus was an important factor for the change of bone mass and architecture [6]. If varus or valgus deformity of the knee joint occurs, it will change the lower limb alignment, as well as local mechanical loading on the tibial plateau, thus results in the joint under abnormal mechanical environment. These changes break the mechanostat of bone tissue. So when knee joint deformity occurs, bone tissue will adjust its structure and bone mineral density (BMD) distribution of proximal tibia to adapt to this abnormal mechanical loading which may be one of the causes of osteoarthritis. Animal experiments proved that the former varus or valgus deformity was associated with the occurrence of knee osteoarthritis [7]. Afterward, some longitudinal studies on patients during early stage osteoarthritis found that if varus or valgus deformity occurred before osteoarthritis, it might be one of the causes of osteoarthritis [8]. As a result, the abnormal loading may be the cause of osteoarthritis [9]. And for patients already with osteoarthritis, knee joint deformity can aggravate the progress of disease.

When varus or valgus deformity of the knee joint happens, the loading acted on the tibial plateau will change and result in changes of BMD in proximal tibia, and it could cause the ratio of medial BMD to lateral BMD at the tibial plateau (M:L BMD Ratio) to become larger or smaller, which can increase the risk of knee osteoarthritis [10]. Thus BMD can be used an index for measuring bone quality [11]. In clinics, radiography is often used to detect BMD, but at least 30 percent change of BMD is required before it can be detectable radiographically [12]. So it is impossible to detect changes of BMD during early osteoarthritis with radiography accurately, nor to predict it. Quantitative bone remodeling theory in combination with finite element method cannot only be used to simulate bone structure efficiently, but also analyze the change of BMD caused by the change of loads and explore the relationship between mechanical environment and osteoarthritis, thus provide theoretical basis for prevention and diagnosis of early osteoarthritis.

The important aspect of bone remodeling simulation is quantitative bone remodeling theory, in which mathematical formula is utilized to quantitatively describe the remodeling process of bone tissue. One of the fundamental theories of bone remodeling is the theory of adaptive elasticity proposed by Cowin et al. [13], Hegedus et al. [14] and Firoozbakhsh et al. [15]. It was based on general continuum mechanics principles. Hart et al. [16] combined this theory with finite element model. Afterwards Weinans et al. [17], Xinghua et al. [18,19] developed the bone density adaptation algorithm, in which the strain energy density (SED) was used as mechanical stimulus and the bone internal structure was described by the apparent density. Another representative quantitative bone remodeling theory was proposed by Huiskes et al. [20]. The behaviors of osteoclasts resorption and osteoblasts formation were separately described. The co-effects of osteoclasts and osteoblasts can simulate the growth, adaptation and maintenance of trabecular [20,21]. With the development of computer technology, the minimum element size of the model used for simulation can be as small as 50 microns. From the previous bone remodeling studies, it is shown that the complex structure of trabecular bone can be simulated [22-24].

Some researchers successfully simulated the change of internal bone structures quantitatively, e.g. Weinans et al. simulated the change of periprosthetic BMD in a two-dimensional (2D) proximal femur with prosthesis and analyzed the effects of different prosthesis materials and fixation methods on distribution of periprosthetic BMD [25]. Similarly, the periprosthetic BMD change of ankle and tibia after ankle arthroplasty were simulated, and bone loss caused by stress shielding was quantitatively analyzed [26]. The periprosthetic BMD in 2D proximal tibia with tibial implant was also simulated and the bone loss caused by four different fixation methods of long stem prosthesis was studied [27]. Later, the BMD change in proximal tibia after long-stemmed total knee arthroplasty with and without bisphosphonates treatment were simulated, and the role of bisphosphonates treatment for mitigating bone loss of proximal tibia was investigated [28]. In addition, Zhu et al. developed a high-order nonlinear equation of bone remodeling, and the internal density distribution and external shape of vertebral body were simulated [18]. The effects of functionally gradient materials on periprosthetic BMD after implanting dental prosthesis were also simulated [29,30]. But these simulations were mostly based on 2D model. Using 2D model to represent three-dimensional (3D) structure has its limitation since 2D model cannot accurately describe the actual structure of bone and its loading condition. So it is very necessary to establish 3D models and simulate internal structures of 3D bone models using quantitative bone remodeling theory in combination with finite element method.

There are also some investigations regarding the simulation of internal structures of 3D bone models. For example, Marsik et al. established a 3D finite element model of proximal femur and simulated its internal structure, and their simulation results were consistent with real proximal femoral structures [31]. 3D finite element models of femur, tibia and mandible were also established to simulate the internal BMD distributions [32]. The effect of different prosthesis fixation methods for periprosthetic bone resorption after total knee arthroplasty with 3D tibial models were simulated and analyzed [33]. Sharma et al. modeled the intact 3D finite element models of glenoid and glenoid prosthesis-bone. They simulated not only the BMD distribution, but also the influences of different materials and fixation methods of prosthesis on periprosthetic BMD [34]. Although some 3D bone remodeling simulations were successful, similar studies on proximal tibia are few. There are even fewer investigations on the relationship between the change of mechanical environment and knee osteoarthritis based on 3D bone remodeling simulation.

Accordingly, this paper aimed to simulate 3D internal structure of proximal tibia under different mechanical environments and to explore the relationship between the change of mechanical environment and the internal structure change of proximal tibia. This study may help to better understand the mechanism of osteoarthritis and provide theoretical basis for early diagnosis and risk prediction for knee osteoarthritis in clinics, thus can serve as theoretical basis for further study on periprosthetic bone resorption after total knee arthroplasty and optimal design of prosthesis.

## Methods

In this paper, the bone remodeling control equation proposed by Weinans et al. [17] in combination with finite element method was utilized to simulate the internal structure of 3D proximal tibia with fixed shape. The load applied on proximal tibia included joint contact force and ligament forces. In order to validate the method in this paper and investigate the influence of mechanical environment on the BMD of proximal tibia, the internal structure of proximal tibia of young people under normal mechanical environment was simulated first. The BMD of the selected regions of interest (ROIs) were obtained and compared with clinical measurements to verify if the method adopted in this paper can accurately simulate BMD distribution of 3D proximal tibia. Then the loading condition on the tibial plateau in valgus knee was changed according to the changing pattern of joint contact force, and the change of BMD in proximal tibia of old people with 6° vaglus deformity was simulated.

### Bone remodeling control equation

The control equation of bone remodeling process is as follows [17]:

(1.1) $$\mathrm{d\rho}/\mathrm{dt}=B\left(U_{a}/\rho -k\right)\;0<\rho \leq \rho_{\mathrm{cb}}$$

(1.2) $$U_{a}=\frac{1}{n}\sum_{i=1}^{n}U_{i}$$

Where *ρ* is apparent density, which is used to describe the characteristics of internal structure of bone. *U*_*a*_ is apparent strain energy density. *U*_*a*_*/ρ* is strain energy density per unit mass, which is used as mechanical stimulus. *B* and *k* are constants. *ρ*_*cb*_ is the maximal bone mineral density, which is usually chosen the density of cortical bone. *U*_*i*_ is apparent strain energy density for loading case *i. n* is the total number of loading cases. In this paper, the constant *B* is taken as 0.05(g/cm^3^)^2^ (MPa × time-unit)^-1^. The reference stimulus *k* is 0.14 J/g. The maximal BMD is 1.92 g/cm^3^ and the Poisson’s ratio is 0.3.

The piecewise function proposed by Zhu et al. is used to describe the relationships between elastic modulus and apparent density according to cancellous bone microstructural models and bone physiology as follows [35]:

(2) $$\begin{array}{l} E=\left\{\begin{array}{cc} 1007\times \rho^{2} & \rho \leq 0.25\\ 255\times \rho & 0.25<\rho \leq 0.4\\ 2972\times \rho^{2}-933\times \rho & 0.4<\rho \leq 1.2\\ 1763\times \rho^{3.2} & \rho >1.2 \end{array}\right) \end{array}$$

Wherein, the units of elastic modulus and apparent density are MPa and g/cm^3^, respectively. This expression is more specific in describing the relationship between apparent density of cancellous bone and elastic modulus.

**Figure 1:**
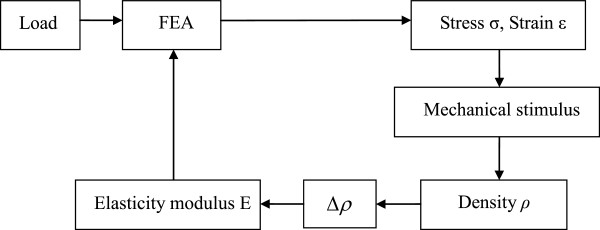
**Iterative feedback mechanism of bone remodeling simulation in combination with finite element method **[36]**.**

The flow chart of bone structure simulation using quantitative bone remodeling theory in combination with finite element method is shown in Figure 1[36].

The iteration will stop when the bone remodeling process reaches equilibrium condition. According to the physiological mechanism of bone remodeling, bone remodeling equilibrium condition is as follows:

(3) $$\mathrm{d\rho}/\mathrm{dt}=0,i.e.U/\rho =k\;\mathrm{or}\;\rho =\rho_{\min}\;\mathrm{or}\;\rho =\rho_{\max}$$

Where *ρ*_min_ is taken as 0.01 g/cm^3^ and *ρ*_max_ = *ρ*_cb_. In this paper *ρ*_cb_ = 1.92 g/cm^3^.

### Finite element model

#### The 3D finite element model of proximal tibia for young normal people

**Figure 2:**
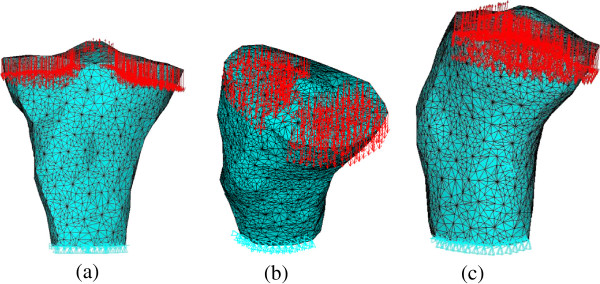
**The 3D finite element model of proximal tibia. (a)** Front view. **(b)** Top view. **(c)** Right view.

The 3D finite element model of proximal tibia for young normal people is derived from the CT scan data of a right proximal tibia of a patient. This patient is male, 34 years old with the height of 173 cm and the body weight of 70 kg. The left tibia of this patient is fractured, but the right knee is healthy. The images were reconstructed into 3D model of proximal tibia in MIMICS software, and then imported into ANSYS software to mesh. The finite element model is shown in Figure 2. This model contains bone tissue in proximal tibia, but the structure of articular cartilage and other soft tissues are not included. This model includes 176708 tetrahedral elements and 30920 nodes. The average element size is 2.0490 mm. It was shown that the initial density had little effect on the simulated results [37]. Thus the initial density is chosen as 0.8 g/cm^3^ in this paper.

The proximal tibia model is constrained distally and the loads applied on it consist of joint contact force, anterior shear force produced by joint contact force, medial collateral ligament force and anterior cruciate ligament force. According to the anatomical structure of knee joint, there are four main ligaments on the knee joint, i.e. medial collateral ligament, lateral collateral ligament, anterior cruciate ligament, and posterior cruciate ligament. In addition to the lateral collateral ligament that is attached to fibula, the other three ligaments are all attached to proximal tibia [38,39]. Shelburne et al. used a 3D model of lower limb to calculate the joint contact force and ligament forces on the normal knee in a complete gait cycle [40]. Posterior cruciate ligament was found unloaded during stance. Thus the ligament forces that applied on proximal tibia in this paper include medial collateral ligament force and anterior cruciate ligament force (see Figure 2). The magnitudes of loads applied on proximal tibia are the mean value of 0%, 10%, 20%, 30%, 40%, 50% and 60% gait cycle loading [40]. The loads applied on the proximal tibia, i.e. joint contact force, shear force, anterior cruciate ligament force and medial collateral ligament force are 1233.3 N, 102.04 N, 139.3 N and 6.4 N, respectively.

The joint contact force acted on tibial plateau, and the medial and lateral tibial plateau bore about 55% and 45% of it [41]. The direction of joint contact force is perpendicular to the surface of meniscus and the part of the tibial plateau not covered by the meniscus. Actually the medial and lateral tibial plateau is covered by medial and lateral meniscus, respectively. The coverage area of medial and lateral tibial plateau by meniscus is about 64% and 84%, respectively, and this proportion remains unchanged during growth [42]. The main role of meniscus is to bear the forces acted on tibial plateau and can carry about 40-60% of the total force [42]. The applied loads of ligaments include the medial collateral ligament force and the anterior cruciate ligament force. The positions of ligament forces acted on proximal tibia are the attached region of ligaments, and the load directions are consistent with ligament orientations [43,44].

#### The 3D finite element model of proximal tibia for old people with 6° valgus deformity

We simulated the internal BMD distribution of proximal tibia with 6° valgus deformity. The reason was that Hulet et al. measured BMD of proximal tibia for the patients who needed treatment due to knee pain, in which 22 cases were valgus deformity of the knee joint and the valgus angle was larger than 4° [12].

The loadings acted on tibial plateau are changed due to the change of lower limb alignment in valgus knees. In normal knees, the connection between femoral head and ankle center is through the center of knee joint, and the loading acted on articular surface are relatively uniform. But when valgus deformity of the knee joint occurred, the lower limb alignment was through lateral tibial plateau, the load center would offset to the lateral tibial plateau and in the corresponding plateau the loading increased [45]. The resultant pressure on medial and lateral plateau in knees with 5° valgus deformity is similar to that in normal knees, while the distribution of loadings acted on the medial and lateral tibial plateau was changed [46]. So in this paper, we assumed that the resultant joint force was unchanged when knee valgus deformity occurred, and the lateral and medial tibial plateau accounted for about 66.5% and 33.5% of joint contact force, respectively. We also simulated the change of internal structure of proximal tibia in valgus knees from uniform initial density. The initial density is 0.8 g/cm^3^.

### BMD measurement

**Figure 3:**
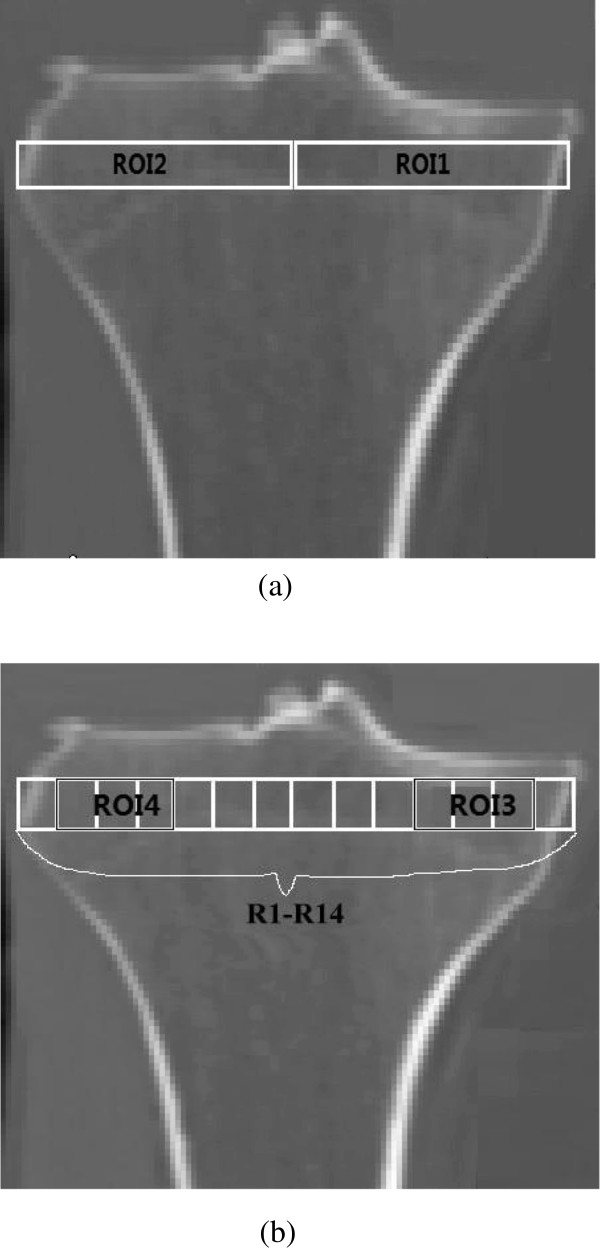
**The coronal diagram of the four selected ROIs according to the measuring regions in clinics. (a)** ROI1 and ROI2. **(b)** ROI3 and ROI4.

In order to verify the simulation method and compare with clinical observations, in this paper, four ROIs in proximal tibia are selected to analyze and compare the BMD. These four ROIs are throughout the tibial plateau from front to back. The selection methods are the same with the actual clinical region selection methods in the literature. The coronal plane diagram for these four selected ROIs of proximal tibia is shown in Figure 3. Figure 3 shows the CT-images of right proximal tibia of a patient, which is used not only to mark the locations of ROIs, but also to compare with the simulated results in Results section. ROI1 and ROI2 are the same as the measuring regions selected by Hudson et al. in clinics [47]. Taken fibular head as reference, the height of ROIs is one-half the distance from the fibular head to the superior border of the cortical plate, and its width is one half of tibial plateau. The ROIs located beneath the medial and lateral tibial plateau are named ROI1 and ROI2, and the coronal plane diagram of these two ROIs is shown in Figure 3a. ROI3 and ROI4 are the same as the measuring regions selected by Hulet et al. in clinics [12]. The distance from the fibular head to the superior border of the cortical plate is about 11 mm in reference [48], while it is 14 mm in our model, the width of tibial plateau is equally divided into 14 parts and these regions are named as R1-R14 successively, where R2, R3, R4 constitute ROI3 in medial tibia, and R11, R12, R13 constitute ROI4 in lateral tibia. The coronal plane diagram of ROI3 and ROI4 is shown in Figure 3b.

## Results and discussion

### Results

**Figure 4:**
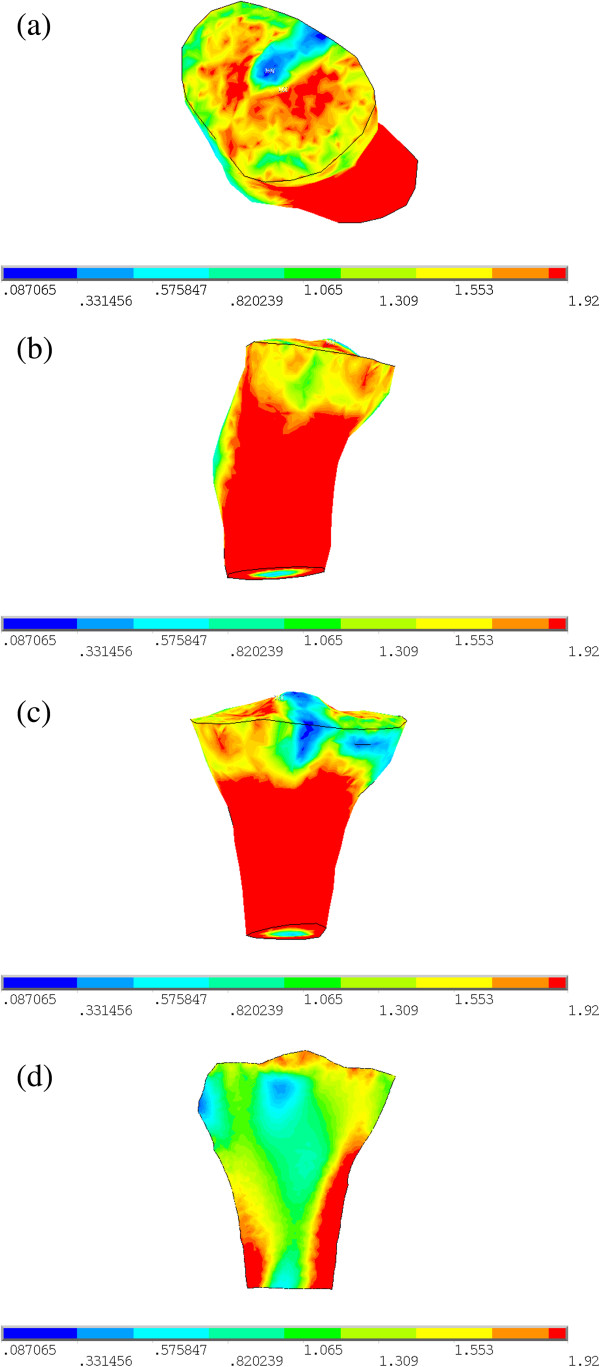
**The simulation result of 3D proximal tibia for young normal people. (a)** Top view. **(b)** Right view. **(c)** Posterior view. **(d)** Cross-sectional view of the coronal plane.

**Figure 5:**
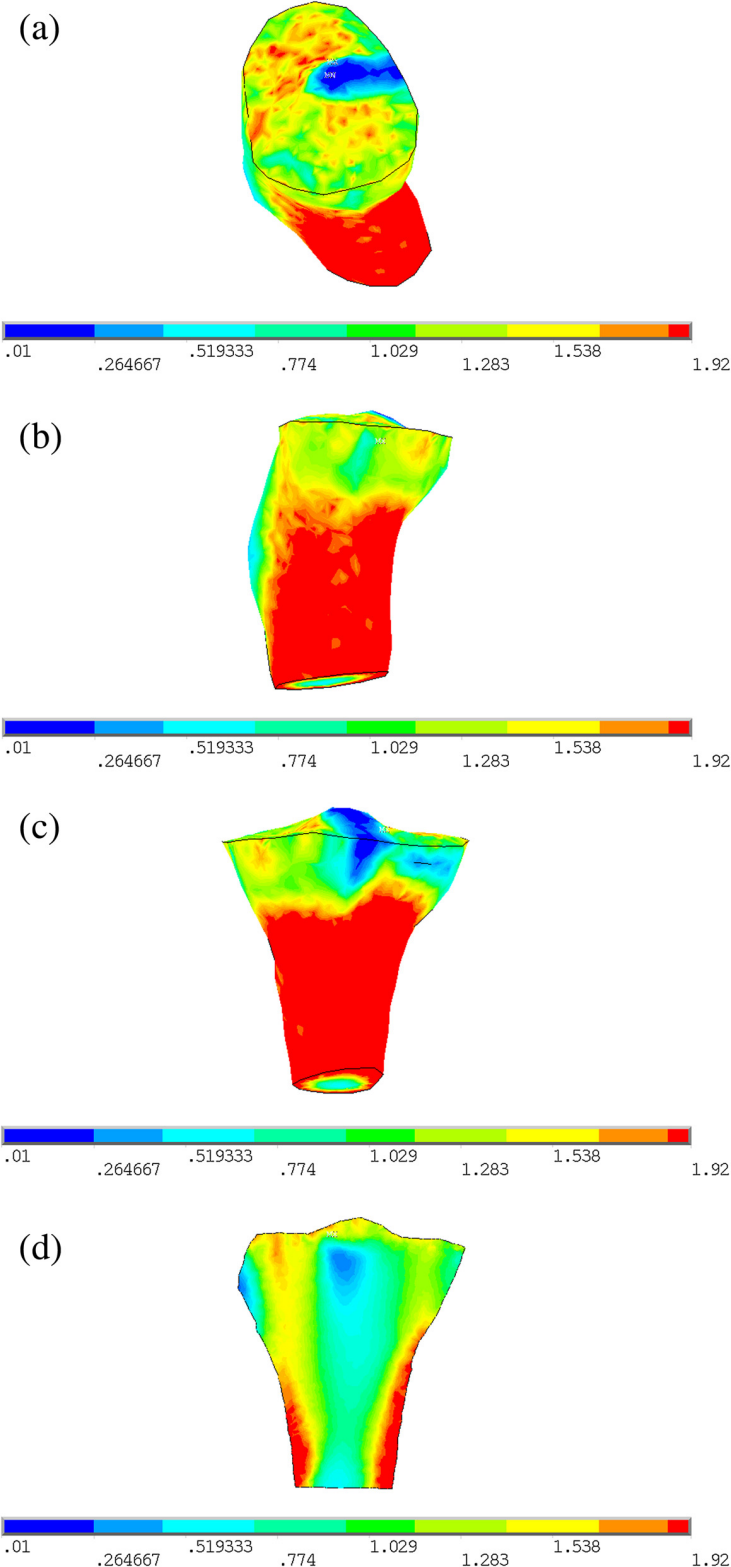
**The simulation result of 3D proximal tibia for old people with 6° valgus deformity. (a)** Top view. **(b)** Right view. **(c)** Posterior view. **(d)** Cross-sectional view of the coronal plane.

Simulation result of the internal structure of 3D proximal tibia for young normal people using quantitative bone remodeling theory in combination with finite element method is shown in Figure 4. Simulation result of internal structure of 3D proximal tibia for old people with 6° valgus deformity is shown in Figure 5.

The simulated normal proximal tibia structure of young people is similar with the real proximal tibia structure (Figure 3), which can be seen from the following three aspects:

(1) Seen from Figure 4a-c, there is a layer of cortical bone covering the whole tibia, and the cortical bone is relatively continuous and completed, This is consistent with the real proximal tibial cortical bone structure in Figure 3;

(2) From Figure 4d it can be seen that there is medullary cavity within distal tibia, This characteristic is also consistent with the real proximal tibial structure in Figure 3;

(3) Beneath the tibial plateau there is cancellous bone, and the BMD in medial region is bigger than that in lateral region. It is in agreement with the measurements in clinics [49].

From the above results it can be seen that the typical characteristics of the simulation results of proximal tibia structure for young normal people are consistent with real tibia.

The average BMDs of the four ROIs and the whole proximal tibia for young normal people and old people with 6° valgus deformity simulation results are listed in Table 1. From the simulation results of proximal tibia of young normal people it can be seen that the average BMDs in ROI1 and ROI2 are 1.23 g/cm^3^ and 1.03 g/cm^3^, and the M:L BMD Ratio is 1.19. While for the same ROIs, the proximal tibial BMDs of 30 young healthy people were measured by Hudson et al. [47], and the obtained average M:L BMD Ratio was 1.20 ± 0.10. Thus it can be seen that the simulated M:L BMD Ratio in this paper is consistent with that measured by Hudson et al. [47]. It shows that the BMD distribution of 3D proximal tibia for young normal people we simulated is consistent with real proximal tibia. Thus it appears that the method used in this paper can simulate and predict the BMD distribution of 3D proximal tibia accurately.

**Table 1:** The average BMD obtained by simulation and measurement in the literature

	**Simulation results of proximal tibia for young normal people**	**Simulation results of proximal tibia for old people with 6° valgus deformity**	**Measurement results of young people obtained by Hudson et al. **[47]**]**	**Measurement results of old people with valgus deformity >4° obtained by Hulet et al. [**[12]**]**
The average BMD of ROI1 (g/cm^3^)	1.23	0.89		
The average BMD of ROI2 (g/cm^3^)	1.03	1.10		
The average BMD of ROI3 (g/cm^3^)	1.246	0.908		0.827 ± 0.198
The average BMD of ROI4 (g/cm^3^)	0.871	1.033		0.939 ± 0.229
The average BMD of the whole (g/cm^3^)	1.343	1.185		
ROI1/ROI2	1.19	0.81	1.20 ± 0.10	
ROI3/ROI4	1.431	0.879		0.878-0.886

The average BMD of young normal people and old people with 6° valgus deformity in the selected ROI and the whole proximal tibia was calculated, and the measurement result in the same zone was listed.

*ROI* region of interest, *BMD* bone mineral density.

From the simulation result of old people with 6° valgus deformity it can be seen that the average BMDs of ROI3 and ROI4, and the M:L BMD Ratio are 0.908 g/cm^3^, 1.033 g/cm^3^ and 0.879, respectively. While the BMDs of proximal tibia of 22 osteoarthritis patients who had severe knee pain with mean age of 71 years old and valgus deformity degree larger than 4° for the same ROIs were measured by Hulet et al. [12] with the average BMDs of ROI3 and ROI4 0.827 ± 0.198 g/cm^3^ and 0.939 ± 0.229 g/cm^3^, and the M:L BMD Ratio 0.878-0.886. It can be seen that under such loading condition, the BMD of medial proximal tibia decreases and that of lateral region increases, i.e. the M:L BMD Ratio is very small. This result is consistent with the clinical observation data of knee osteoarthritis patients with valgus deformity, which means that when valgus deformity of the knee occurs, the BMD distribution of proximal tibia changes accordingly under that abnormal mechanical environment for a long period.

## Discussion

In this paper, the internal structure of 3D proximal tibia was simulated using bone remodeling theory in combination with finite element method. The internal structure of 3D proximal tibia of young normal people was simulated to validate the method used in this paper. Comparing the simulated results in Table 1 with the observations in the literature, it can be seen that the average BMD ratio of ROI1 to ROI2 in the simulated result of internal structure of 3D proximal tibia for young normal people is consistent with the result of young healthy people in the same area measured by Hudson et al. [47]. Hence with the method used in this paper the BMD distribution of 3D proximal tibia can be simulated more accurately.

After the simulated results were verified, the loading applied on the proximal tibia was changed according to the changing pattern of loading when valgus deformity occurred. Then the simulated result of internal structure of proximal tibia for old people with 6° vaglus deformity was obtained. The average BMD ratio of ROI3 to ROI4 was in the range that measured in the same region of old people with greater than 4° vaglus deformity who need treatment because of knee joint pain [12]. It means that when the loads change, the internal structure of proximal tibia is consistent with clinical observations on osteoarthritis patients, that is, under the mechanical environment of valgus knees, the BMD distribution of proximal tibia has changed. It shows that mechanical environment changes the BMD distribution under cartilage in proximal tibia, and the change of mechanical environment is one of the causes for bone structural abnormity. If knee joint deformity occurred, it may lead to the change of loads acted on the tibial plateau, and that change can break the primary mechanostat, which will enhance the bone remodeling of proximal tibia and increase the risk of osteoarthritis.

In clinics, the bone mass and distribution of cancellous bone in proximal tibia are thought to be closely associated with age and gender, and BMD of proximal tibia would reduce with ageing for males, as well as females [50]. In Table 1, the BMDs of ROI1, ROI3 and the whole proximal tibia obtained in our simulations for young normal people are all greater than old people with valgus deformity. The BMDs of ROI1, ROI3 and the whole decrease by 27.64%, 27.127% and 11.765% for old people in comparison with those of young people. These results show that with ageing, the BMD of proximal tibia became smaller. This pattern is consistent with clinical observations. But in ROI2 and ROI4, however, the average BMD of young normal people is smaller than old people with valgus deformity. The reason may be that we simulated the BMD distribution of proximal tibia in this paper when valgus deformity of the knee occurs, the joint contact force is not changed in valgus knees, but its distribution changes with the load center on tibial plateau moving from medial to lateral region [45], resulting in lateral tibial plateau bears more load, and the BMDs of ROI2 and ROI4 beneath lateral tibial plateau increase. Even so, the average BMD of proximal tibia of old people in ROI2 and ROI4 is still smaller than the whole BMD of proximal tibia of young people; the BMD changing pattern is in agreement with the loading that acted on tibial plateau. In addition, from Figures 4d and 5d we can see that the cortical thickness of proximal tibia of young normal people is thicker than that of old people with 6° valgus deformity.

The specific features of osteoarthritis such as joint space narrowing, osteophyte and hardening are closely related to extreme M:L BMD Ratio, and the M:L BMD Ratio is proportional to medial joint space narrowing and inversely proportional to lateral joint space narrowing [10]. If M:L BMD Ratio is too low, it can increase the possibility of lateral joint space narrowing, lateral osteophyte formation and hardening. When the varus or valgus deformity of the knee joint occurs, the axial alignment of the lower extremity changes and load center will be moved from the center of tibial plateau to medial or lateral tibial plateau [45]. This changes the distribution of loads acted on tibial plateau, thus affects the internal structure of proximal tibia, i.e. BMD of the proximal tibia would increase with the increasing load on its corresponding plateau, which leads to the change of M:L BMD Ratio. Thus the M:L BMD Ratio can be used as a diagnosis index for osteoarthritis in clinics. Another direct application of the method used in this paper is to accurately simulate the change of BMD of proximal tibia caused by load changes when varus or valgus deformity occurred, so it can provide theoretical basis for prevention and early diagnosis of osteoarthritis.

In summary, the change of BMD in proximal tibia is closely related to mechanical environment, and mechanical environment is the direct reason for the change of internal structure of proximal tibia. If keen joint abnormalities occur, being under this abnormal mechanical environment for a long period may lead to osteoarthritis. The proximal tibial model we used in this study didn’t contain the articular cartilage. That is why the degeneration of articular cartilage was not discussed. But the effect of changes of mechanical properties of articular cartilage was included in the change of loading. In this paper the relationship between mechanical environment and bone morphological structure is mainly studied. Although the effect of articular cartilage cannot be investigated directly, it was shown that the subchondral bone structure and the degree of articular cartilage damage were closely related [51]. The change of BMD occurred after valgus deformity may imply that the articular cartilage has been damaged already and moreover, the damage of articular cartilage will cause the uneven loading distribution on tibial plateau, which will aggravate the change of subchondral bone. From the above result it can be seen, the change of mechanical environment directly lead to the change of bone morphological structure. The mechanism of osteoarthritis is analyzed from the influence of mechanical environment on bone structure, which helps to better understand the mechanism of osteoarthritis.

In order to verify the convergence of the model utilized in this paper (model 1), the same proximal tibia was also meshed with a smaller or a larger element size, i.e. the average element size of this two model is 1.6000 mm (model 2) and 2.6706 mm (model 3), respectively, while the average element size of model 1 used in this paper is 2.0490 mm. These three models contain 176708, 360789 and 80353 tetrahedral elements, respectively. Under the same loading and boundary conditions, these three models were analyzed in ANSYS software. The detailed information and results are listed in Table 2. It is found that the relative difference of average element equivalent stress, average equivalent strain and average SED between model 1 and model 2 are 0.01296%, 1.4467% and 4.5333%. Although the relative difference of element size between these two models is 28.0625%, all the relative differences in finite element results are within 5%. The differences in results are relative small, but decreasing the element size will cause the number of elements increasing and consuming too much time to compute. Comparing the finite element results of model 3 with model 1, the relative differences are 9.5115%, 7.9421% and 14.6909%, respectively. It is shown that the relative differences between model 3 and model 1 are large. From the above analyses it is shown that the element size of the model used in this study is small enough to guarantee the accuracy of the results.

**Table 2:** The detailed information and results of three finite element models with different meshes

	**Model 1**	**Model 2**	**Model 3**	**Relative difference between model 1 and model 2 (%)**	**Relative difference between model 1 and model 3 (%)**
Average element size (mm)	2.0490	1.6000	2.6706		
Number of elements	176708	360789	80353		
Average element equivalent stress (MPa)	0.7717	0.7716	0.8451	0.01296	9.5115
Average element equivalent strain	0.0006774	0.0006676	0.0007312	1.4467	7.9421
Average element SED (MPa)	0.0004125	0.0003938	0.0004731	4.5333	14.6909

*SED* strain energy density.

The simulation method in this paper has wide applications. For example, it can be used for further computational simulation on the change of periprosthetic BMD due to stress shielding after total knee arthroplasty or other joint replacement. And the method can also simulate the change of periprosthetic bone mass for different prosthesis material or fixation methods, which provides a theoretical basis for the optimal design of prosthesis material and suitable selection of prosthesis fixation methods. By changing the control equations of bone remodeling process and introducing biological factors, the change of BMD for menopausal women may be further simulated. The method used in this paper can quantitatively simulate the BMD change due to the change of mechanical environment accurately and rapidly. It also solves the problem in clinics that too much time cost on observing the change of BMD in human body, and makes up the disadvantage that some experimental methods cannot be applied directly to human body.

## Conclusions

Bone morphological abnormity is closely related to its abnormal mechanical environment, thus the knee joint deformity would be the cause of osteoarthritis, and the method used in this paper can be used as an effective method to predict the BMD change of proximal tibia under different mechanical environments to help diagnose osteoarthritis in clinics. Another important conclusion is that the M:L BMD Ratio can be used as another criteria to measure the structural change caused by osteoarthritis on specific site.

## Abbreviations

3D: Three-dimensional; 2D: Two-dimensional; BMD: Bone mineral density; ROI: Region of interest; M:L BMD Ratio: The ratio of medial BMD to lateral BMD at the tibial plateau.

## Competing interests

The authors declare that they have no competing interests.

## Authors’ contributions

JF established the finite element model, carried out the numerical simulations and drafted the manuscript. HG conceived of the study design and helped to draft the manuscript. LYK participated in the numerical simulations. DZ provided the CT images. All authors read and approved the final manuscript.
